# MANdatory - why men need (and are needed for) gender equality progress

**DOI:** 10.3389/fpsyg.2024.1263313

**Published:** 2024-03-01

**Authors:** Colette Van Laar, Aster Van Rossum, Natasza Kosakowska-Berezecka, Renata Bongiorno, Katharina Block

**Affiliations:** ^1^Department of Psychology, KU Leuven, Leuven, Belgium; ^2^Fonds Wetenschappelijk Onderzoek, Brussels, Belgium; ^3^Institute of Psychology, University of Gdansk, Gdansk, Pomeranian Voivodeship, Poland; ^4^School of Social Sciences, Bath Spa University, Bath, United Kingdom; ^5^Department of Psychology, University of Amsterdam, Amsterdam, Netherlands

**Keywords:** gender, social equality, social change, men and masculinity, gender roles, precarious manhood

## Abstract

While much progress has been made towards gender equality, diversity and inclusion in the workplace, education and society, recent years have also revealed continuing challenges that slow or halt this progress. To date, the majority of gender equality action has tended to approach gender equality from one side: being focused on the need to remove barriers for girls and women. We argue that this is only half the battle, and that a focus on men is MANdatory, highlighting three key areas: First, we review men’s privileged status as being potentially threatened by progress in gender equality, and the effects of these threats for how men engage in gender-equality progress. Second, we highlight how men themselves are victims of restrictive gender roles, and the consequences of this for men’s physical and mental health, and for their engagement at work and at home. Third, we review the role of men as allies in the fight for gender equality, and on the factors that impede and may aid in increasing men’s involvement. We end with recommendations for work organizations, educational institutions and society at large to reach and involve men as positive agents of social change.

## Introduction

While much progress has been made towards gender equality, diversity and inclusion, recent years have also revealed continuing challenges that slow or halt this progress. For example, the covid-19 pandemic has revealed and increased gender inequality (Fisher et al., 2020; Yerkes et al., 2020); the MeToo movement has shone a light on still persistent sexual harassment at work (see Keplinger et al., 2019; Lisnek et al., 2022 for discussions); abortion has now been newly banned or restricted in several EU countries and US states, and austerity policies following the global financial crisis have hollowed out social services supporting gender equality, including access to affordable childcare, housing, and legal services. Indeed, the UN (2022) concluded that if the current rate continues it will take close to 300 years to achieve full gender equality.

We posit that we should not tackle such challenges without rethinking how gender equality is approached, for whom it is beneficial, and what mechanisms are responsible for its slow or stalled progress. To date, most gender equality practitioners, policy makers and researchers have approached gender equality from one side: focused on the removal of barriers for girls and women, and to create organizations, structures and societies allowing girls and women to thrive and succeed - especially in traditionally male-dominated spaces. We aim to show that this is only half the battle: Existing gender inequalities result from the multifaceted nature of gendered power dynamics in various areas of life where women and men are interdependent and play key roles in maintaining or changing the existing status quo.

Much of the research we review here is based on a western binary view of gender, where people are defined (both by others and by themselves) as either women or men. We fully acknowledge that the gender binary is a social construct and does not reflect how a growing number of people define themselves and others (e.g., non-binary, gender fluid, etc., see Hyde et al., 2019). While challenging the gender binary is an important part of change, here we focus on progress towards gender equality as it relates to challenging restrictive traditional gender roles for women and men (girls and boys). That is, we focus on understanding how to remove the pervasive power of gender stereotypes that prescribe and proscribe the gender norms women and men are held to and hold to. We argue that while men’s adherence to masculine norms is a large part of the problem, men are and should also be a large part of the solution, and that the improvement of the situation for women (and men, and nonbinary individuals) depends on men. Paradoxically then our goal is to show that barriers for women will not be removed without removing gender-restrictive barriers for men, and that gender equality will not be achieved without providing men - as well as women and those who identify as non-binary - true freedom from the pervasive power of gender stereotypes. In examining men’s roles we of course recognize the tremendous heterogeneity and intersectionality within men, and that many men are not necessarily privileged in terms of ethnicity, social class, physical ability or sexual orientation (Coston and Kimmel, 2012).

In this review we highlight men’s roles in gender equality in three ways: First, we focus on how men’s privileged higher status is threatened by gender equality progress, and consequences of this threat for gender-equality initiatives. Specifically, although women comprise half the world’s population, men continue to have more power than women. Existing hierarchies and inequities also mean that men may perceive women’s gains – in politics, education and work – as a threat to men’s status. We explain how withdrawing support for gender equality helps men maintain their advantageous position in the gender hierarchy and restores their threatened manhood status. We describe how gendered hierarchies and gender inequities are maintained by cultural ideologies that justify and rationalize men’s power over women, and discuss research on precarious manhood and zero-sum beliefs - plus their links to men’s reluctance to support gender equality. We note that understanding these threats and their consequences is an important step in addressing gender equality in a potentially more inclusive and effective way.

Second, we focus on men as themselves falling victim to restrictive gender roles. We argue that despite their dominance in the hierarchy, existing gender roles can also affect men’s ability to thrive and do well in education, work and social life. Men continue to be under pressure to uphold unrealistic and unhealthy expectations about ideal or ‘real’ manhood, and we show how such expectations affect men and others in various ways: They encourage men to engage in risky behaviors and aggression and prevent men from taking care of their mental and physical health. Also, they create masculinity contest cultures in organizations, and strong work devotion in men that may both harm men’s health and wellbeing, and lead men to shy away from positive caring roles known to benefit the self and others, such as caring roles in education and health care, and for children and others at home.

Third, we focus on the importance of men as allies in gender equality progress: on how men have been stepping up alongside women to make a difference, and how their investments are critical for gender equality progress. We discuss factors that can contribute to men recognizing the problem of sexism - including interventions that encourage emotional empathy for women as targets of sexism and reduce empathy towards men as perpetrators. We further discuss factors that may encourage men to become involved in change, such as how feminist men are portrayed, whether movement norms are inclusive of men’s involvement, and women’s reactions to men’s ally behaviors.

We conclude with men as pivotal agents for change: those who have power to make a difference in work organizations, educational institutions, and society.

## The current status of gender equality and men as agents within this

Over the past few decades much research in social psychology, sociology, business studies and organizational psychology has addressed diversity and inclusion by focusing on the representation and involvement of women in work, education and society. This important research has documented women’s underrepresentation in key domains generally, and in traditionally male domains and at higher levels of organizations and society more specifically. Much attention has been focused on understanding the mechanisms that maintain and can reduce this underrepresentation. For example, the mechanisms that lead to lower selection of women job candidates, that lower the likelihood of women’s promotion, and that increase the likelihood women will exit organizations or occupational domains. This research shows that women face more lack-of-fit and prejudice; less welcoming social climates, plus hostility and sexual harassment, that lead them to feel a lower sense of belonging in work and education (Eagly and Karau, 2002; Berdahl, 2007; Heilman, 2012). Further, this research highlights the impact of women’s care roles on their work involvement and ways in which motherhood is associated with disadvantages at work (Barnett et al., 2004; Cuddy et al., 2004; Williams et al., 2016). Traditionally, social scientists have focused on ways to rectify these issues as ways of increasing gender equality.

Undoubtedly, these endeavors have at least partially succeeded (UN, 2022): We have made considerable progress in some areas, with women (at least in the global North) increasingly represented in work: working more hours, in more domains, and at more and also higher levels of organizations and society. Nevertheless, the progress has been partial and despite considerable efforts we are a long way from gender being irrelevant to work, educational and health outcomes. Gender continues to be highly predictive of the domains in which people work, how much they work, their status in organizations as well as their salary (Vuorinen-Lampila, 2016; Blau and Kahn, 2017; Dämmrich and Blossfeld, 2017). Indeed, organizations report difficulties reaching their gender equality goals, despite strong motivation and effort - including various programs, changes in formal policies, opportunities and training regarding diversity, equality, and inclusion (DEI) (Dover et al., 2016b, 2020a; Saba et al., 2021). Moreover, there are considerable differences in gender equality across countries. For example, in the EU Sweden scores 84 on the Gender Equality Index, whilst Greece scores at a 53 (European Institute for Gender Equality, 2022). In the wider world, even greater disparities exist, with Rwanda having closed 79% of its overall gender gap whilst Afghanistan still has the global worst scores of 41% gender parity (World Economic Forum, 2023).

Such persisting gender inequality is not only at odds with the goals most democratic societies strive for and with UN Developmental Goals (UN, 2015), but also has direct negative impacts on lives. For example, women remain much more economically dependent on others than are men, and this lack of independence has serious consequences for women and children when women are or become single or single parents (Malone et al., 2010; Gonçalves et al., 2021). Moreover, the continuing inequality means societies do not benefit from the full range of talent and qualities women can contribute. In the meantime, not only women and minority gender groups are disadvantaged: It is becoming increasingly clear that men are also negatively impacted by strong gender roles and inequities, for example in their health, well-being and social relationships, and in opportunities to connect with their children (Croft et al., 2015; Meeussen et al., 2020; Van Rossum et al., 2024). Children meanwhile are denied access to their fathers, with increasing research showing negative consequences of this low involvement (Amato and Rivera, 1999; Aldous and Mulligan, 2002; Fletcher, 2011; Croft et al., 2014; Opondo et al., 2016; Rollè et al., 2019; Cano and Hofmeister, 2023).

We argue that continuing to singularly focus on women no longer optimally serves progress towards gender equality. Rather, broadening our perspective to bring men’s role into focus is now needed: We below outline the different ways in which a focus on men can help us understand and advance gender equality progress.

## Gender equality progress as a potential threat to men

To date, women have been the driving force of gender equality strategies and struggles (Holter, 2014). Data from 34 countries show that women place more importance on gender equality than men, and that they are less optimistic about the likelihood of attaining gender equality (Pew Research Center, 2020). Compared with men, women declare a stronger willingness to support gender-related collective actions (data from 42 countries, Kosakowska-Berezecka et al., 2020); devote more time to foster DEI within organizations (Women in the Workplace Report, 2022); and more often actively participate in promoting gender equality (Radke et al., 2016). Although men often report favorable attitudes toward gender equality, they are also reluctant to support policy initiatives, and to feel that gender equality has already been achieved (Levtov et al., 2014; DIT, 2023).

While there is a strong and successful history of men’s allyship in gender equality progress (we return to this in section three), below we shed light on three underlying mechanisms explaining why some men are either not allies, or actively resist DEI programs. First, we focus on men’s perception of gender equality progress as achieved at the expense of men. Then, we discuss the role of strong legitimizing beliefs leaving men less likely to recognize women’s unfair treatment. And finally we describe how, on an individual and deeper level, prescriptive and proscriptive masculine norms present in our societies, and the precarious nature of manhood fuel men’s resistance. While some of these mechanisms are specific to gender (e.g., the precarious nature of masculinity in response to gender change), other mechanisms are relevant more generally in understanding why men - as an advantaged group in most contexts - might resist general diversity change and pro-minority inclusion, including for example resistance to the inclusion of those with different ethnic backgrounds, or challenges to the status quo more generally.

### Women’s gains = men’s losses

One of the underlying mechanisms explaining men’s ambivalence can be related to the fact that as the higher-status group in society, men might be seen as having more to lose than to gain from gender equality. Men universally tend to have more agency and power than women: making more money and holding higher power positions in most countries (Global Gender Gap Report, 2022). When analyzing gender equality progress, it is crucial to understand that collective action by less privileged groups (such as women) is likely to highlight the unfair privilege of high-status ones (here men). This, in turn can trigger the need in men to legitimize their higher status (Sidanius and Pratto, 1999; Leach et al., 2002; Iyer and Leach, 2009).

In general, people like to see the sociopolitical contexts that favor their ingroup as fair and just (Cichocka and Jost, 2014). Changes to the existing economic or political hierarchies may be stressful and perceived as threatening, especially to those with the most to lose (Scheepers and Ellemers, 2018). As such, men as the high-status group may be especially motivated to defend the status quo, and manifest their resistance to gender equality actions both openly and more subtly (Osborne et al., 2019). Some men may view women’s advances at work as threatening to men’s power, and may thus see women as usurpers of male power and as men’s competitors (Fiske and Taylor, 2013). Such a mindset, which is referred to as the “belief in a zero-sum game,” can lead men to believe that more power and money for women means less power and money for men (*cf.* Ruthig et al., 2017; Kosakowska-Berezecka et al., 2020). Evidence indeed shows that men show stronger belief in this “zero-sum game” than women, and generally view gender relations through a more threatening and competitive lens (Bosson et al., 2012; Wilkins et al., 2015; Kuchynka et al., 2018). As DEI policies target the gender hierarchy, men may think that they have more to lose, both materially (“women will take over our positions, jobs, money”) but also symbolically (“women will challenge traditional men’s beliefs and values”) (Stephan and Stephan, 2000). As a result, some men, especially those with higher gender identification (Maass et al., 2003), may feel they are themselves victims of discrimination, and manifest defensive responses to status threats (see also DIT, 2023). Such a response was voiced in 2023 by Chemistry Noble Laurate Kurt Wüthrich, who warned against “discrimination against men” in STEM fields resulting from (in his perception) too much focus on DEI measures (Heidt, 2023). Affirmative action encouraging the selection of women candidates, rewarding teams hiring ethnic minorities, or highlighting women’s success more than men’s may then be perceived as directly harming men. Indeed, there is evidence that men’s zero-sum thinking increases after reminders of women’s societal status gains (Kuchynka et al., 2018), and that men viewed decreases in discrimination against women as directly linked with increases in discrimination against men (Kehn and Ruthig, 2013). Not surprisingly, zero-sum beliefs can then fuel hostility towards women in positions of power: Indeed, recent research shows that men endorsing zero-sum beliefs about gender were more inclined to endorse hostile sexism against women, which in turn reduced men’s gender equality support (Ruthig et al., 2017; Kosakowska-Berezecka et al., 2020).

Counter-intuitively, such resistance may be especially present in contexts in which gender equality is perceived as (increasingly) valued and where DEI actions are perceived as (becoming) successful. A recent cross-cultural study indeed shows that men manifest lower support for gender equality actions in countries with higher gender equality levels (where DEI programs are more prevalent), and that this lower support may in turn stall gender equality progress (Kosakowska-Berezecka et al., 2020). This translates to organizations as well: if men perceive DEI messages as more robust and as favoring women over men, they might reduce their support for DEI actions. There is evidence showing that when exposed to diversity statements, advantaged groups (e.g., White men) were more likely to view their group as disadvantaged, and manifest cardiovascular reactions signalizing threat (Dover et al., 2016a,b, 2020b). Practitioners should thus be aware that programs promoting DEI can be challenged by some men who feel threatened and see themselves as victims rather than beneficiaries, and that effectively managing threat reactions is likely to strengthen program effectiveness.

### Blindness of the privileged

Apart from perceiving gender equality progress as benefiting women at the expense of men, another potential mechanism underlying men’s resistance is linked to the fact that - on average - men are less likely than women to recognize unfair treatment of women (Drury and Kaiser, 2014). Men find it harder than women to detect discriminatory acts (Swim et al., 2001), to recognize derogatory statements about women as prejudiced (Rodin et al., 1990), and to notice unfavorable employment practices that disadvantage women (Blodorn et al., 2012; DIT, 2023). Men may have even more trouble detecting discrimination if it is manifested in a subtler form of paternalistic and benevolent acts, as they may see these as well-intentioned and harmless forms of support and protection favoring women (Glick et al., 2000; Gervais et al., 2010; Becker and Swim, 2011). Pratto and Stewart (2012) address this issue even more broadly by pointing out a wider cultural phenomenon also for other social inequalities (e.g., based on ethnicity): noting the acceptance of social inequality linked with the implicit assumption that the dominance of a group is normal. Thus, men might not recognize their status as advantageous, as it is culturally considered default, and this disguises their privileged position as “normal” while perpetuating stereotypes and maintaining the lower position of other groups. Additionally, men, as a dominant group, can be more inclined to promote their power, and as hierarchy-enhancing discrimination is often institutionalized, no individual effort is necessary to maintain men’s group dominance (Pratto and Stewart, 2012). Men’s lack of recognition of their privilege and their lower sensitivity towards subtle forms of discrimination poses a difficult barrier for gender equality progress as it lowers the likelihood that men will oppose such more subtle and derogating forms of discrimination, and can decrease men’s willingness to support change (Ellemers and Barreto, 2009; Becker and Wright, 2011; Van Laar et al., 2019).

The “blindness” men can face to recognize unequal treatment of women is linked to the fact that men are also more prone than women to endorse meritocratic-type beliefs that individuals are responsible for their life successes, and that life outcomes are purely the result of one’s efforts and achievements. At the same time, men are more likely to neglect structural barriers and pervasive gender stereotypes that contribute to status differences faced by women (Jost et al., 2004). Indeed, men show stronger legitimizing beliefs, such as the belief in individual mobility (i.e., the belief that regardless of one’s group membership one can achieve merit-based success; Major et al., 2002), stronger social dominance orientation (support for social hierarchy and acceptance of superiority of some groups over others, Sidanius and Pratto, 1999), and stronger beliefs that their high status is earned (Sidanius and Pratto, 1999). Such legitimizing beliefs help men rationalize their privileged status, and to perceive less privileged groups (such as women) as not having worked hard enough. Perceiving the existing social hierarchy as fair, legitimate and well-deserved allows men to maintain the status quo, and their own psychological and moral comfort (Jost et al., 2004). Recognizing the structural barriers and status hierarchy as unfair to women would force men into a potentially unpleasant realization that they do not deserve their personal or group status (Adams et al., 2006). Not being fully aware of their privileged status, and failing to recognize when and why discrimination happens, men may thus find it hard to be DEI allies.

However, seeing only women and not men as the victims of these processes is a fallacy. Even though men tend to have more power than women, men’s decisions and behaviors are also restricted by social and cultural expectations related to masculinity (we return to these issues in the section on men themselves as victims of restrictive gender roles).

### Male identity and precarious manhood

A third and potential deeper mechanism underlying men’s resistance to support gender equality is the nature of male identity and the potential perceived precariousness of that identity. On one hand, men have more power than women: greater control over the creation, distribution of, and access to resources (which predicts their safety, health, freedom and quality of life, e.g., Rivers and Josephs, 2010). Also, men’s greater size and thus strength makes them more apt to take power by force; and finally, there are numerous beliefs permeating social life that maintain and legitimize the higher status of men over women (Pratto and Walker, 2004; Alesina et al., 2013). Indeed, hierarchies and gender inequities are maintained and reinforced by gender differences in resource control and physical strength, and by cultural ideologies that justify and rationalize men’s power over women (Pratto and Walker, 2004). Such a high place in the hierarchy, however, also leaves men vulnerable to having to prove this status (Bosson et al., 2022).

Although men have greater structural power than women in most cultures, the nature of manhood (relative to womanhood) in most societies today is precarious, it is “hard won and can be easily lost” (Vandello et al., 2008; Bosson et al., 2022). In order to prove their higher status, men need to consistently demonstrate agency and dominance, and avoid femininity to garner respect. As the value of being seen as manly is high, and femininity is valued less, gender prescriptions and proscriptions are endorsed more strongly for men than women (Bosson et al., 2022), and when their masculinity is threatened men are inclined to take actions to restore their masculinity. There is growing evidence that manhood is threatened by for example making men appear feminine (Kosakowska-Berezecka et al., 2016a,b) and that this can lead to a wide array of compensatory behaviors, including aggression (Bosson et al., 2009); harassment of women (Maass et al., 2003); financial risk-taking (Weaver et al., 2013); avoidance of feminine behaviors (Rudman and Mescher, 2013); and manifesting greater liking for prototypical compared to non-prototypical men (Schmitt and Branscombe, 2001). Men may face masculinity threats as a result of engaging in DEI efforts: For example, as gender equality is often seen as a “women’s issue” (Kaufman, 2004) men can be hesitant to support it because they fear such opinions or actions might make them appear less masculine. The term “feminist man” is often associated with traits considered anti-masculine, non-attractive, and low in potency (Anderson, 2009), as well as linked with femininity, weakness and homosexuality (Rudman et al., 2012). Research has shown that such labels can have consequences for men’s willingness to support gender equality– when actions are described as “feminist” (vs. without that label) they are less likely to be supported by men (Conlin and Heesacker, 2018). Defensive reactions to threatened masculinity may also increase men’s prejudice towards women and minority groups (Glick et al., 2007; Weaver and Vescio, 2015; Alonso, 2018; Ching, 2021; Wellman et al., 2021; Vallerga and Zurbriggen, 2022), increase denial of discrimination against women (Weaver and Vescio, 2015), and decrease men’s support for and participation in DEI initiatives (Kosakowska-Berezecka et al., 2016a). Men who endorse masculine work ideals may feel that diversity and inclusion put their privileged masculine status at risk (Dover et al., 2016a) further reducing their interest in DEI policies (Hill, 2009; Marchlewska et al., 2021).

Presumably, withdrawing support for gender equality helps men restore their threatened manhood status and maintain their position in the gender hierarchy (Herek, 1986; Sidanius and Pratto, 1999; Vandello and Bosson, 2013; Kosakowska-Berezecka et al., 2016b). Similar threat reactions are observed in other high-status groups, for example when white Americans are informed that by 2050 minority Americans will outnumber non-Hispanic white Americans (Craig and Richeson, 2014). Research has shown that white individuals who are made aware of this experience more anger and fear toward minorities, express more explicit and implicit anti-outgroup attitudes, and show greater support for anti-minority policies (Craig and Richeson, 2014; for similar results in Canada, United Kingdom and United States see Stefaniak and Wohl, 2021). Masculine threats and need for compensatory actions to regain power posit an important barrier for gaining acceptance and support for DEI. The need to compensate for masculinity loss experienced by men who endorse precarious manhood beliefs can thus backfire on DEI programs. As such, perceiving DEI policies as targeting men’s privilege and as aiming to change the status quo at the expense of men is an important challenge that practitioners cannot afford to neglect.

Taken together, DEI programs may never be fully successful as long as they are perceived as focused on women (or minorities in general) only. As long as gender equality is seen as progressing at the expense of men, men may resist gender equality and measures by withdrawing support, or by actively protesting against DEI actions. One of the most crucial and promising questions therefore is to understand when and how men can perceive gender equality as beneficial for them. There is a robust evidence showing that men do gain from gender equality in terms of health, well-being, and their overall happiness, as we discuss in the next section.

## Men themselves as victims of restrictive gender roles

Most attention in research and public debate has focused on the negative consequences of gender roles and stereotypes for women. In no way do we as the authors minimize the myriad of hardships women face because of gender inequality. However, we make the case that these hardships are also in part the result of our failure to consider the effects of restrictive gender norms for men, and that an examination of the complete set of processes is needed to adequately address gender inequality, and to include men in overcoming gender inequality.

Substantial research shows the pervasive restrictions that gender roles impose on men. First, traditional views on masculinity discourage men to care for their physical and mental health, and encourage dangerous and risky behavior, leading to worldwide gender discrepancies in health outcomes and longevity (Brannon, 1976; Courtenay, 2000; WHO Regional Office for Europe, 2018; Vandello et al., 2022). Second, men are still commonly expected to be ambitious, successful and devoted to their work, which creates unhealthy pressure and hinders men’s domestic engagement (Berdahl et al., 2018). Third, it is still often disapproved for men to show interest in traditionally feminine domains, such as childcare and HEED occupations (Healthcare, Early Education and Domestic domains – Croft et al., 2015), while such interest is known to benefit men’s wellbeing and women’s position in society (Meeussen et al., 2020). Below, we discuss gender role restrictions for men in each of these three domains: men’s health and well-being, workplace masculinity norms, and domestic engagement and HEED interests, and argue that bringing attention to these processes is necessary to engage men in the pursuit of gender equality.

### Risks to men’s health and wellbeing

#### Physical health and risk behavior

Across the world, men have a lower life expectancy than women (OECD and European Union, 2020; WHO, 2020). Among the leading causes of men’s premature death are life-style related conditions such as cancer, cardiovascular disease and respiratory illnesses (WHO Regional Office for Europe, 2018). Health behaviors that may in part cause such conditions are displayed more by men than women: consuming alcohol (Erol and Karpyak, 2015), eating meat (Stoll-Kleemann and Schmidt, 2017), and smoking (WHO, 2022); and these health behaviors are predicted by men’s endorsement of and adherence to traditional views on masculinity (Mahalik et al., 2007; Iwamoto et al., 2011; Iwamoto and Smiler, 2013; Roberts et al., 2014; Houle et al., 2015; Wilkinson et al., 2018; Rosenfeld and Tomiyama, 2021). Indeed, research has suggested that certain unhealthy behaviors are seen as a ‘sign’ of masculinity (Nichter et al., 2006; Vartanian et al., 2007; de Visser and McDonnell, 2012; Vartanian, 2015), and that men may thus choose unhealthy behaviors to prove their masculine status, and to fit prevailing gender norms (Chiou et al., 2013; Fugitt and Ham, 2018; Nakagawa and Hart, 2019; Mesler et al., 2022). Importantly, men are also less likely to consult a doctor when they experience pain or are ill (European Commission, 2011). Traditional masculinity norms are at odds with help-seeking, as men are expected to be self-reliant and discouraged from showing weakness or being overly emotional (Prentice and Carranza, 2002). A systematic literature review of 41 papers has indeed identified masculinity norms that present barriers in men’s help-seeking, such as need for independence and control, restricted emotional expression, and embarrassment (Yousaf et al., 2015a). Also, especially men who more strongly attach their self-worth to how well they live up to masculine expectations report inhibitions against and delays in seeking healthcare (Himmelstein and Sanchez, 2016).

Another major cause of premature death for men is non-intentional injuries (WHO Regional Office for Europe, 2018). This again has been tied to gender roles: Men have been found to overall take more risks than women (Byrnes et al., 1999; Dohmen et al., 2011; Breivik et al., 2017), and risk taking is more appreciated for men as it conveys courage and toughness (Bosson et al., 2009; Fowler and Geers, 2017). This however can come at high cost for men’s own wellbeing and that of others’, as reflected in the higher incidences for men of traffic accidents (WHO, 2021a), drug-related deaths (European Monitoring Centre for Drugs and Drug Addiction, 2022), sports injuries (National Safety Council, 2022), and incarceration (Federal Bureau of Prisons, 2023). Risk-taking is a way to protect or prove one’s status as a ‘real man’ (Vandello et al., 2008; Giaccardi et al., 2017), for example through aggression (Bosson et al., 2009; Braly et al., 2018; Borgogna et al., 2022) and risky financial decisions (Weaver et al., 2013; Parent et al., 2018). Men who feel distressed about not meeting masculinity standards reported more assaults causing injuries and armed assaults (Reidy et al., 2016a), and reported engaging in more risky sexual behavior (Reidy et al., 2016b). Moreover, men whose masculinity was threatened showed higher pain tolerance (Berke et al., 2017), suggesting another pathway through which precarious manhood may lead to health risks: by overstepping one’s own physical boundaries. Also cross-nationally, recent findings showed that country-level variations in precarious manhood beliefs predict men’s risky health behaviors - such as transportation accidents and contact with venomous animals (Vandello et al., 2022). Indeed, in countries where precarious manhood beliefs are more prevalent, men’s life expectancy is shorter by 6 years (Vandello et al., 2022).

#### Mental health

Men’s mental health also shows detrimental effects of male gender roles. Research showed that adherence to traditional masculinity norms relates to poorer mental health (Wong et al., 2017), higher suicidal ideation (Coleman, 2015; King et al., 2020) and later suicide (Coleman et al., 2020). Worldwide, men commit suicide more than twice as often as women (WHO, 2021b) and in 2021 almost 80% of US suicides were committed by men (Centers for Disease Control and Prevention, 2023). Keeping others at a distance may be an important factor in the negative relation between masculine gender norms and men’s mental health. Indeed, recent research shows that boys and men are generally more socially isolated than girls and women (Way, 2013; Umberson et al., 2022), which could form a major health and mortality risk for men (Holt-Lunstad et al., 2010). Attempts to meet masculine expectations may stand in men’s way towards close connections, social support, and if needed, professional help. For example, after a gender threat, men reported lower closeness and commitment to their romantic partner to re-establish their masculine status (Lamarche et al., 2021). Also, as with medical care, men less often seek psychological help than women (Möller-Leimkühler, 2002; Milner et al., 2020), and especially when they endorse traditional masculine ideals (Berger et al., 2005; Vogel et al., 2011; Yousaf et al., 2015b), adhere to masculine norms such as self-reliance and emotional control (Mahalik and Di Bianca, 2021), and self-stigmatize seeking help (e.g., feel seeking help threatens self-esteem; Vogel et al., 2011; Mahalik and Di Bianca, 2021). Such issues may also be of particular consequence for trans-individuals and those who consider themselves nonbinary: mental wellbeing is significantly more vulnerable in these individuals (Timmins et al., 2017; Newcomb et al., 2020; Puckett et al., 2020), and male roles and prescriptions to avoid seeking help may not aid in addressing any mental health issues.

Men who have attempted suicide described inhibitions against expressing negative emotions to others and not being quite able to identify or to put into words their feelings and emotional pain (Cleary, 2012). Such trouble identifying and describing own emotions - or *alexithymia* - is associated with depression (Li et al., 2015) and is more prevalent among men (Levant et al., 2009). Researchers have argued that as a result of gender socialization, some men show a mild form of alexithymia normative for the male gender role (i.e., normative male alexithymia; Levant et al., 2006). Importantly, this mild form is related to lower psychological wellbeing, reduced social relationship quality, and fear of relational intimacy (Karakis and Levant, 2012; Guvensel et al., 2018). Men may indeed fear expressing intimacy as research shows this can put them at risk for social rejection and negative evaluations, especially from other men (Gaia, 2013).

These severe consequences for men’s physical and mental wellbeing tend to stay under the radar and are not sufficiently addressed in societal conversations and policy making. Better understanding and acknowledgment of these processes is crucial also to increase men’s awareness about the personal benefits of gender equality and what is in fact at stake for them (Holter, 2014), and likely will also motivate men more as allies in gender equality progress.

### Pitfalls of masculinity contests in the workplace

Constraining masculinity norms are also at play at work, as shown by research on “masculinity contests” and “work devotion norms.” Masculinity contests refer to organizational environments that require employees (men, women and other) to prove their adherence to masculine work ideals (Berdahl et al., 2018). These ideals require employees to avoid showing weakness and seeking support, and to instead display strength and endurance, prioritize work, evidencing a strongly competitive mindset (Glick et al., 2018). Such organizational environments cultivate work devotion norms encouraging employees to dedicate high time and effort to work, for example through overtime and pushing to meet deadlines (Williams, 2000; Blair-Loy, 2001). Working part-time is looked down upon, which can create obstacles for employees to engage in domestic work or childcare, and to achieve work-family balance. Masculinity contests are (perceived as) more prevalent in male-dominated organizations (Glick et al., 2018; Munsch et al., 2018). For example, in stereotypically masculine fields such as academics and STEM (Cooper, 2000; Damaske et al., 2014) working overtime is often glorified, and seen as endurance and toughness (e.g., people showing off their exhaustion; Cooper, 2000) whereas seeking flexibility is stigmatized (Williams et al., 2013).

Such masculinity contest and work devotion norms may detrimentally affect employees’ wellbeing. For example, masculinity contest at work are related to lower general (Glick et al., 2018) and psychological health (e.g., increased stress levels and burnout; Glick et al., 2018; Matos et al., 2018; Rawski and Workman-Stark, 2018; Workman-Stark, 2021). In addition, organizational cultures characterized by masculinity contests are related to increased imposter feelings and lower belonging (Vial et al., 2022), increased turnover intentions (Glick et al., 2018; Matos et al., 2018; Rawski and Workman-Stark, 2018; Workman-Stark, 2021), and poorer work-life balance (Glick et al., 2018; Matos et al., 2018). Such a restrictive and competitive work culture mirroring the masculine gender role is not only detrimental to members of groups that are typically excluded and discriminated by such a discourse (e.g., women and ethnic-, cultural-, or gender minority groups), but also for (heterosexual) men who are expected to fit well with and thrive under these norms. For instance, research has shown how hyper-masculine occupational stereotypes (e.g., in the military) may discourage not only women but also men who feel they do not fit this stereotypical ‘macho’ image (Peters et al., 2015). In addition, these contests may be particularly difficult for trans-individuals, those who identify as nonbinary, or who do not fit easily in the gender binary categorization (Köllen, 2016).

Besides these negative health and wellbeing consequences of masculinity contest and work devotion norms that affect everyone, there are also specific repercussions harming men. For example, research has shown that working men who adhere more (vs. less) to traditional masculinity norms rated their own overall wellbeing and the wellbeing of other traditional working men as lower (Kim et al., 2020). Moreover, men who fail to meet or actively resist masculine work standards not only violate work norms, but also gender norms - thereby risking backlash through social rejection and work-related sanctions (Burke and Black, 1997). For example, Moss-Racusin et al. (2010b) showed that men applicants for a manager position who defied gender norms by being modest were perceived as weaker and less agentic, and were less liked than modest woman applicants. Similarly, men who applied for an internal promotion and were described as advocates for their team (instead of for themselves) were estimated as less agentic and competent, and more recommended to be released from the company, compared to similar women (Bosak et al., 2018). Moreover, men leaders who sought more help (vs. less) were rated as less competent, while there was no such difference for women leaders (Rosette et al., 2015). These findings show how men may face significant dilemmas: possible harm to their health and wellbeing if they adhere to masculine work norms, but risking social and work-related backlash if they do not.

Restrictive masculinity norms in the workplace not only harm individuals’ wellbeing but can also obstruct efforts to create a more diverse and inclusive workplace. For instance, in organizations where masculinity contest norms are more prevalent, employees report more sexism and sexual and ethnic harassment (Glick et al., 2018; Kuchynka et al., 2018). Furthermore, in such environments masculine status may be especially precarious and easily threatened (Berdahl et al., 2018). Importantly, as noted earlier, research shows that defensive reactions to threatened masculinity may increase men’s prejudice towards women and minority groups (Glick et al., 2007; Weaver and Vescio, 2015; Alonso, 2018; Ching, 2021; Wellman et al., 2021) and decrease men’s support for and participation in DEI (Kosakowska-Berezecka et al., 2016a). Yet again, this shows the importance of considering the restrictions posed by masculinity norms, for the sake of both men’s wellbeing and gender equality at large.

### Underrepresentation of men in domestic and HEED roles

A third domain in which men face gender role restrictions is in domestic engagement, and more generally, representation in HEED domains (Health care, Elementary Education and the Domestic sphere - Croft et al., 2015; Meeussen et al., 2020). While women have increasingly moved toward traditionally masculine domains (e.g., STEM fields, management positions) men are still underrepresented in traditionally feminine (HEED) domains. Across the world there are substantially fewer men in traditionally feminine occupations, for example with men being only 33% of the primary education teachers worldwide (World Bank, 2023) and 24% of the human health workers in the EU (European Institute for Gender Equality, 2023). Men also continue to engage less in housework and childcare than women (US Bureau of Labor Statistics, 2023). For instance, European men spent on average about 21 h a week on childcare (vs. 39 h by women -European Institute for Gender Equality, 2020). Such traditionally feminine roles typically build on a communal orientation, which refers to being warm, empathic and caring towards others (Bakan, 1966). Even though these roles are often still devalued relative to traditionally masculine roles (Block et al., 2018), adopting a communal orientation has been shown to be good for people’s relationships and wellbeing (Carlson et al., 2016; Kosakowska-Berezecka et al., 2016b; Le et al., 2018; Petts and Knoester, 2020). For example, people with more communal values report higher life satisfaction and more positive emotions (Hofer et al., 2006; Sheldon and Cooper, 2008; Le et al., 2013), and US men (and women) expect higher wellbeing should paternity leave become paid (Moss-Racusin et al., 2021).

One reason for the persisting underrepresentation of men in HEED is that gender associations linking men to agency and women to communion are generally internalized (see Croft et al., 2015). According to gender norms it is both typical and desirable for men to be agentic and for women to be communal (Heilman, 2001; Prentice and Carranza, 2002; Bosson et al., 2022). Recent research shows that especially this association between women and communion has increased over the years, and that it is stronger than the association between men and agency (Eagly et al., 2020). These gender norms become part of people’s self-concept early in life, e.g., through parents’ and others’ socializing behavior (Edwards et al., 2003; Martin and Ruble, 2010) and may steer boys’ and men’s interests away from HEED (Chaffee and Plante, 2022).

Secondly, men’s communal engagement may be hindered by external barriers (see Croft et al., 2015 and Meeussen et al., 2020 for reviews). Men who do have a traditionally feminine occupation may experience a conflict between their work identity (requiring communality) and their identity as a man (requiring agency; Cross and Bagilhole, 2002; Simpson, 2005), which could reduce their wellbeing (Wolfram et al., 2009). In order to protect their masculine identity against threats, men may indeed turn away from HEED roles (Chaffee et al., 2020; Kaplan and Offer, 2022). Not only may men themselves choose HEED roles less in order to avoid gender role conflict and masculinity threat, but men are also sometimes directly discouraged from making such choices. For example, mothers may discourage fathers from getting more involved in childcare and housework, as they believe that men are less skilled in that regard, and as they seek to affirm their own identity as a mother (i.e., maternal gatekeeping; Allen and Hawkins, 1999; McBride et al., 2005; Gaunt, 2008; Gaunt and Pinho, 2018; Meeussen and Van Laar, 2018; Bareket et al., 2020). In addition, men who do show communal involvement may receive backlash from others. For instance – and as noted earlier - men who seek flexibility arrangements at work have been found to be evaluated more negatively (Vandello et al., 2013), and at risk for work-related sanctions (Rudman and Mescher, 2013). Similarly, negative evaluations may occur for men in stereotypically feminine professions such as early education, or positions aimed at fostering interpersonal relationships at work (Heilman and Wallen, 2010; Moss-Racusin and Johnson, 2016; Halper et al., 2019).

Importantly, besides benefits for their own personal wellbeing, men taking up more communal roles would also promote more gender equality at work (Croft et al., 2015; Meeussen et al., 2020; Reverberi et al., 2021). Since women still take up most of the housework and childcare (e.g., the percentage of stay-at-home mothers in the US was almost four times that of stay-at-home dads, Livingston, 2018; and in parts of Europe 70% of women work part-time, compared to only 28% of men, CBS, 2022) there would be more opportunities for women in heterosexual couples to pursue a work career if men were to take up more housework and childcare (Meeussen et al., 2019; Moss-Racusin et al., 2021). Importantly, research suggests there is pluralistic ignorance among men about having communal values, with men overestimating how much their peers endorse a traditional view of men as agentic rather than communal – which in turn has negative consequences for their own involvement (Van Grootel et al., 2018). It is therefore of great importance to break this misconception and to bring people’s attention - and especially men’s attention- to the value of cultivating a stronger sense of communality. Indeed, there are signs that men may be moving in this direction, as for example a majority of interviewed men in academia expressed wanting to be more involved at home and reported making efforts to do so (Damaske et al., 2014). Research has moreover shown that highly educated and career-driven women find communally oriented men more attractive than men who are not (Meeussen et al., 2019), suggesting norms may indeed be changing at least in some (often leading) subsections of society.

In conclusion, men’s contributions to gender equality can then also be increased through involvement in domestic and more general HEED domains. Paired with attention to other ways in which men are negatively restricted through gender roles (e.g., in their health and wellbeing, in their strong devotion to unhealthy work environments) such a focus on gender restrictions for men, and their effects on men, women, other gender groups, and their children can help pave the way for men’s involvement in gender equality. Indeed in the next section we consider men’s role as allies in gender equality progress.

## Men as allies in gender equality progress

The importance of mobilizing men to advance gender equality has become a topic of increasing focus (Kimmel et al., 2004; Flood, 2011). As highlighted in earlier sections of this paper, women - the disadvantaged/low power group with the most to gain from challenging gender inequality - have historically been at the center of gender movement theorizing and research (see Maddison and Sawer, 2013; Radke et al., 2016). Yet there is increasing recognition that achieving positive and sustainable change requires a change to men’s attitudes and behavior at the interpersonal and intergroup level (Mahalik et al., 2003; Locke and Mahalik, 2005; Parrott, 2009; Fox and Tang, 2014; Croft et al., 2015; Meeussen et al., 2020); along with changes to the broader systems and processes over which men still preside that maintain their power and privilege. First, we discuss men’s orientation to gender equality and gender equality movements. Second and third, we discuss factors that aid and may interfere with men’s advocacy for gender equality.

### Changing men’s attitudes towards gender equality and gender equality movements

The involvement of men as allies for gender equality is not new - there is a long history of men being willing to confront sexism. For example, in the early twentieth century, the US Men’s League for Women’s Suffrage provided critical support to the women’s suffrage movement, including speeches, fundraising and lobbying government officials (Kroeger, 2017). During the second wave of feminism in the 1970s, anti-sexist men’s groups - such as Men Against Patriarchy (MOP) - emerged in Australia to support the women’s cause (Flood, 2014). Today through international organizations such as The White Ribbon Campaign, He For She (UN), and the MenEngage Alliance, a small but growing number of men around the world are becoming involved in gender equality activism, including the prevention of violence against women (Flood, 2014). Particularly active in gender change are trans-men and those who identify as non-binary, who themselves fight daily against restrictive gender norms.

Changes in men’s attitudes over time have also been encouraged and inspired by worldwide feminist movements, and their accompanying changes in gender relations at home and work. Research shows that men’s attitudes towards feminism and gender equality have become more progressive over time as the feminist movement has provided women greater rights and freedoms (Bolzendahl and Myers, 2004; Donnelly et al., 2016; Scarborough et al., 2019). Moreover, research has found men’s exposure to feminism through awareness raising/education, and through feminist exemplars in their lives, an important determinant of men’s feminist attitudes. For instance, using nationlly representative US data (1974–1998), Bolzendahl and Myers (2004) showed that having a female spouse in the labor force was the most important determinant of men’s feminist attitudes in the four areas examined (abortion, pre-marital sex, gender-roles, and family responsibilities,). In addition, more highly educated men, and with more highly educated mothers, were also more likely to have feminist attitudes (also see Stoltenberg, 1990; Casey and Smith, 2010).

Yet, support for gender equality has not fully taken root among men. While surveys tend to show a steady increase in men’s support (but see important nuances, e.g., Levtov et al., 2014; DIT, 2023), only a minority of men self-identify as feminist (Silver et al., 2019). This too is tied to social norms: men’s feminist identification and activism is influenced by norms surrounding men’s participation (see Kutlaca et al., 2022) as well as by portrayals of feminist men. For instance, Wiley et al. (2013) found that men’s feminist identification and willingness to engage in gender-related collective action was greatest when feminist men were portrayed positively, and when men’s involvement was considered necessary for progress. This was in comparison to conditions where men’s involvement was depicted as a barrier to progress, and to a neutral control condition where a history of feminism was described without mentioning men. Below we discuss the factors that may aid and that may prevent men from involvement in gender equality progress.

### Factors that may aid men’s involvement in gender equality progress

The above findings provide inroads to compel men’s support for gender equality. We below discuss several leverages for change that can aid men’s support: explicit encouragement of men’s involvement; positive contact with feminists; raising awareness about the costs of masculinity for men; and more generally appealing to men’s group-based and personal interests; encouraging empathy for women as targets of sexism and reducing empathy for men as perpetrators.

#### Encouragement of men’s involvement

Explicit encouragement of men’s involvement may be an important factor in engaging men in gender-related change. For example, Sherf et al. (2017) found that more explicit encouragement of men to partake in workplace gender-equality initiatives can have positive effects. This is because men’s low involvement can be due to a perceived lack of psychological standing, or perceived low legitimacy to act on behalf of this cause. In this research, participants were asked to volunteer to be part of a companywide taskforce on gender parity (the control condition) and some participants also received information that the CEO had made a request for both men and women volunteers. As expected, this additional information provided a boost to men’s volunteerism, increasing to 55% (vs. 33% in the control condition without a specific invitation for men’s participation).

#### Positive contact with feminists

Inspired by intergroup contact theory (Allport, 1954; Pettigrew and Tropp, 2006; Wiley et al., 2021) showed that men’s positive contact with feminists can also facilitate support. In two studies (one cross-sectional, one half longitudinal), straight men living in the US were asked to indicate the extent to which they had had positive contact with feminist women (interactions that made them feel “accepted,” “supported” and “welcomed”). Participants were also asked to indicate how much solidarity they felt with the feminist cause, their public and domestic support for gender equality, and their awareness of gender privilege (i.e., that men are afforded greater opportunities because they are men). Across studies, those who reported more positive contact with feminists also reported more solidarity with feminists. Solidarity with feminists was in turn positively associated with greater awareness of gender privilege. However, only in the cross-sectional study was men’s solidarity with feminism also positively associated with public and domestic support for gender equality. Nonetheless, the benefits of positive contact with feminist women to men’s solidarity, and in turn their gender privilege awareness, points to a potential important avenue to aid men’s involvement (for related findings, see Case et al., 2014; Vázquez et al., 2021; Wu et al., 2024).

#### Programs that raise awareness about the costs of masculinity for men

Programs promoting awareness about the costs of masculinity for men may also increase men’s involvement, with as prime example programs on health and well-being. It is notable, for example, that none of the wellbeing programs targeting boys and young men reviewed in a recent meta-analysis by Gwyther et al. (2019) incorporated masculinity as a framework with which to understand and address mental-health issues. Men’s adherence to (some) masculine norms can be damaging, not only to women and the gender-equality cause, but also to the physical and mental health of men. This was one important conclusion of Wong et al. (2017) meta-analysis on masculinity and mental-health outcomes incorporating 78 studies with almost 20,000 mostly White, heterosexual US men. In included studies, participants were asked about their conformity to up to eleven different masculine norms, along with assessment of positive mental health (e.g., life satisfaction) and negative mental health (e.g., depression). Men’s adherence to three norms in particular: power over women (desire to dominate women); playboy (desiring multiple, non-committed sexual relationships); and self-reliance (unwillingness to seek help), the first two of which were strongly associated with sexism, and were significantly, robustly, and unfavorably associated with all mental-health outcomes. In part these factors may make it more difficult for men to have positive relationships with women, with this in turn leading to lower mental health (Wong et al., 2016). Educating men and boys on the benefits of rejecting unhealthy masculine norms may therefore be a promising avenue to boost men’s support for gender equality (see, e.g., Case et al., 2014; Lux et al., 2024; Equimundo).^1^

#### Appealing to men’s group-based interests

More generally, research has found men to be more likely to participate in gender equality initiatives when they are framed to appeal to men’s group-based interests, such as greater access to paid parental leave or greater workplace flexibility for men. For instance, Farrell et al. (2021) examined support for gender equality initiatives amongst STEM faculty members. Initiatives framed as benefiting men and women, (vs. just women), received more support from men by reducing their program fairness concerns, and increasing their internal motivation to engage. There is also some evidence that leaders who frame gender equality as a common cause for men and women (vs. a women’s issue) can facilitate men’s engagement (Hardacre and Subašić, 2018; Subašić et al., 2018).

It follows that men would be motivated to support action framed as consistent with their group-based interests and/or of benefit to men and women. However, this focus may also be counter-productive to the extent that it normalizes men’s engagement only in circumstances where men stand to visibly benefit. For many gender-inequality issues, men’s engagement is needed, even and perhaps especially, when change requires removing their group-based privileges, and/or challenging problematic behaviors and systemic factors that help maintain men’s privilege. For instance, in the case of challenging men’s violence against women, change requires confronting victim-blaming narratives and organizational responses that protect men accused of wrongdoing from accountability (Bergman et al., 2002; McDonald, 2012).

#### Encouraging empathy for women as targets and reducing empathy for men as perpetrators

Increasing men’s empathy for women as targets of sexism and gender-based violence may also be effective in increasing men’s support for gender-related social change. This focus may make men more empathetic to women facing sexism and gender-based violence, but also to men and non-binary people as victims. As highlighted above, there are numerous examples of men supporting feminism and participating in gender equality initiatives out of a concern for justice for women (e.g., to prevent men’s violence against women) rather than self-interest. Experiences that prompt men to feel empathy for women targets of sexism are likely to be important to this. For example, Becker and Swim (2011) conducted a diary study whereby men were asked to consider the frequency of sexist incidents experienced by women. Men in an “empathy inducement” condition were also asked to consider how the women targets of sexism felt. The empathy inducement was critical to producing a significant reduction in men’s endorsement of sexist beliefs. Other research looking at sexual violence and rape myth acceptance (i.e., men’s greater tendency to blame victims/survivors and downplay negative effects) has also found that empathy interventions with men that described men as the victim/survivors of sexual assault increased men’s empathy for, and reduced rape myth acceptance, when it came to women victim/survivors (Foubert and Newberry, 2006; Stewart, 2014). Also, Mazzuca and colleagues showed that as men experienced more relative deprivation on behalf of women they were more motivated to engage in gender equality collective action at work, with this mediated by increased guilt about gender inequalities and decreased fear of backlash, plus the moral conviction of acting for gender equality (Mazzuca et al., 2022).

In addition to increasing men’s empathy for women who suffer sexism, research by Bongiorno et al. (2020) has shown that reducing men’s empathy for men accused of sexism may also be important. In this research, participants read about a young woman student sexually harassed by a man student. Men reported higher victim blaming than women (consistent with previous research), and men’s greater empathy than women for the man accused fully explained this gender difference in victim blaming. Men’s and women’s empathy for the complainant was high overall and did not differ significantly. In a second study, Bongiorno et al. (2020) found that both men’s greater empathy for the accused and victim blaming could be reduced by having men consider how the sexual harassment affected the complainant’s (vs. the accused’s) life. Moreover, both lesser empathy for the accused and greater empathy for the complainant were important in explaining lower victim-blaming in the complainant (vs. accused) -perspective-taking condition.

Bongiorno and colleagues’ research indeed shows that men may be more prone to excusing men’s wrongdoing than women because they are more likely to focus on the perspectives and feelings of men accused of sexism. Yet when prompted to consider the perspectives and feelings of the women men on average respond in more prosocial and less sexist ways. Other research has highlighted how men’s involvement with gender-equality advocacy out of a concern for justice for women is linked to a focus on women’s perspectives. For instance, Casey and Smith (2010) interviewed 27 men involved in programs to end men’s domestic or sexual violence against women. Amongst the three factors critical to men’s involvement was having “sensitizing” experiences, such as hearing first-hand accounts from women on the reality of violent victimization. More generally, experiences of feeling devalued may aid men in taking others’ perspective with devalued identities (see Moss-Racusin et al., 2010a).

### Factors that stand in the way of men’s advocacy for gender related social change

The above research highlights key factors known to be related to men’s positive engagement with the promotion of gender equality. Yet, it is important to acknowledge that there is still much in men’s social environments that works against their positive engagement. This includes, for example, media focuses on men’s perspectives, including the foregrounding of the plight of men accused of wrongdoing rather than those victimized by men (Meyers, 1996; Kahlor and Eastin, 2011); and gender-segregated networks that provide men more ready access to the perspectives of men accused of sexism (McDonald, 2012). We discuss these below. Also, we consider lip-service to gender-related change, and the benefits and downsides of including men as allies.

#### Biased media

Much of media that individuals, including men, consume (e.g., news, movies, TV shows, video games) is owned, produced, directed, and/or reported by men (or those who work for men; Women’s Media Centre, 2021). This has led to narratives that create and help to reproduce gender inequality, as men’s experiences and perspectives tend to be prioritized, often in ways that serve men’s interests. For example, myths about rape are common in mainstream media reporting, including the myth that women are most likely to be raped by a man stranger in a dark alley (rather than by a man they know), or that women who are raped while under the influence of alcohol are partly responsible (see Tranchese, 2019). There are concerted efforts by feminists to tackle this media bias, including through social media (see Rentschler, 2014) and broader efforts to diversify media to better represent the perspectives, experiences and realities of women (e.g., see BBC’s 50:50 equality project).^2^ However, until this media landscape is changed, men’s exposure to narratives that challenge dominant interpretations serving their interests will remain elusive. This may in turn prevent the widespread development of understandings that could build men’s solidarity with the gender equality cause.

#### Gendered-segregated networks

Another factor that can stand in the way of men’s understanding of and advocacy for gender equality is gender segregation as an ongoing feature of social life, including at work and in friendships (Mehta and Strough, 2009). Indeed, outside family relationships and heterosexual intimate partners (discussed above as an avenue for men’s positive gender equality engagement), much of social life is gender segregated, keeping men from developing friendships and comparisons with women that could promote a better understanding of women’s perspectives and experiences (see also Major, 1994). Feminist theorists have argued that in patriarchal cultures, the domination of women by men is sanctioned and promoted through bonds between men (see Sedgwick, 1985). Flood’s (2008) research highlights how such bonds can shape deeply problematic attitudes and behaviors towards women. Ultimately then, challenging the development and normalization of gender-segregated social networks is an integral part of the change to facilitate men’s support for gender equality. Promising research by Hilliard and Liben (2010) has shown that de-emphasising gender in US preschools (e.g., avoiding gendered language to describe children) does lead to a reduction in children’s gender stereotyped attitudes, and importantly, their preference to only play with same-gender classmates.

#### Lip service to gender-related change

In addition, men’s advocacy for gender equality, when it does happen, is not always based on good intentions or the right approach. Men’s advocacy may be motivated by a desire to boost personal reputation, public or company profile, rather than out of a genuine commitment for change. Referred to as “performative allyship” this is where easy and costless actions are taken by men that look good on the surface, and benefit reputation, but can cost the movement because an appearance of change replaces actual change (Kutlaca et al., 2022). There is also increasing evidence showing further negative consequences of such lip-service to gender-related change (Bromley and Powell, 2012; Bourke et al., 2017; Mor Barak et al., 2021; Baker et al., 2023).

#### Considering effects of the involvement of men in gender advocacy

In considering the engagement of men allies though, it is also important consider potential inadvertent effects. On the one hand men advocates for gender equality are likely to receive more recognition for their work and may have bigger, quicker wins by virtue of their greater access to power and influence (Connell, 2003). Men may be more effective gender advocates because they are perceived as more credible and considered less motivated by self-interest than women (Czopp and Monteith, 2003; Roden et al., 2021). Men’s greater traction as advocates – especially men in significant positions of power and influence – underscores the importance of their engagement. On the other hand, gender inequality of influence within the movement is also an aspect of gender injustice that, if not challenged, can generate resentment from women that forms barriers to effective collaboration (Flood, 2011). Related to this, some men’s engagement may also intentionally or unintentionally reproduce gendered dominance/subordinate relationships (see also Good et al., 2018; Estevan-Reina et al., 2020 for examples when men confront sexism through a paternalistic duty to protect). For instance, Macomber’s (2012) research on “engaging men” groups found that some men would dominate interactions and make claims to expert knowledge in areas they knew little about. Research by Piccigallo et al. (2012) examining men’s participation in anti-rape groups on campus also found some men to be more focused on and affected by men’s than women’s evaluations. In related areas, Bridges (2010) presented a case study of men protesting violence against women through performances of drag at “Walk a Mile in Her Shoes” marches. They observed that because the use of drag by men was derisive, it was ultimately reinforcing, rather than challenging, of gender inequalities.

How men’s participation in gender-equality efforts affects women and their engagement is also an important consideration. In Sherf et al. (2017) research, the impact of a CEO inviting men and women to partake in a taskforce on gender parity (vs. an invitation with no explicit mention of men or women) led to 10% fewer women volunteering. Iyer and Achia (2021) also found that a gender-equality organization described as having a leadership team with a majority (vs. minority) of men reduced women’s collective action intentions via reduced hope and reduced perceptions that the leaders had sufficient awareness of gender inequality. Research by Kutlaca et al. (2022) showed that men’s equal participation with women in gender inequality protests– in comparison to women-only protests –increased women’s identification with the movement only if men played a supportive (vs. leadership) role. Other research by Droogendyk et al. (2016) indeed highlighted that to be good allies, men must consider the challenges of their participation, including the harm if men’s perspectives and feelings are privileged. It is important then in considering men’s ally behavior to understand both the goals this allyship has, and the effects of men’s allyship on other men, as well as on women and others.

## Conclusion: men as agents for change

In the current paper we have provided an overview of why men are needed – and themselves need – gender-related change. While much gender-equality effort focuses on women, we argue that not only are men needed for gender-equality progress to be successful, but that gender restrictions also have significant underexamined effects on men. This new attention towards men will also increase the likelihood that gender-equality efforts will engage men, as it makes clearer what all have to gain. In such endeavors, it is important not to lose sight of the goal: to benefit all, not just those groups or individuals directly affected by specific measures.

We focused on men’s role in gender equality progress in three key ways (see Table 1 for an overview): First, on men’s privileged status as potentially threatened by gender equality progress: how women’s gains may seem men’s losses, how being privileged may lead one to be blind to the disadvantage of others, and how the precarious nature of male identity may make it entertaining gender-related change difficult for men. Second, we focused on men as themselves victims of restrictive roles, and the consequences for men’s physical and mental health, for their engagement at work and at home, and in communal HEED domains in broader society. Third, we considered the men’s role as allies: what is currently known about men’s attitudes to and involvement in gender-related social change, about the factors that impede and may aid in increasing involvement, and about the benefits as well as potential drawbacks of how male engagement is secured.

**Table 1:** Overview of the key factors playing a role in involving men in gender equality progress.

Men’s privileged higher status as threatened by gender equality progress	Consequences for: Cultural ideologies that justify and rationalize men’s power over women. Perception of women’s gains as men’s losses. Blindness of the privileged. Male identity and precarious manhood. Restoration of status through withdrawal of support for gender equality.	Factors that may aid men’s involvement in gender equality progress: Socialization among men more free of gender-related expectations and restrictions. Providing identity safe environments, alleviating precariousness of masculinity and concerns for masculine identity. Raising awareness among men about negative consequences/restrictions of gender norms for men themselves. Lowering pluralistic ignorance among men as to men’s attitudes towards communal traits and roles. Education to raise awareness and knowledge among men of gender-bias, the workings of gender bias and gender-related processes. Promoting more gender diverse networks allowing men to come into direct contact with those who experience gender-related disadvantages and increase their perspective taking and empathy to victims of discrimination. Encouragement of men’s involvement in gender equality progress, also by showcasing male agents and champions support DEI initiatives. Appealing to men’s group-based interests in non-damaging ways (e.g., through appeals to shared common cause). Moving media attention for the plight of men accused of gender-related wrongdoing onto those who have been victimized. Factors that may dampen effects of men’s involvement in gender equality progress: Presenting men as singular culprits of existing gender inequalities. Lip service to gender-related change; wrong motivations for involvement in gender-related change. Ineffective involvement of men in gender equality efforts (reproduction of paternalistic or status/dominance relations).
Men as themselves victims of restrictive gender roles	Consequences for: Men’s physical health and risk behavior. Men’s mental health and wellbeing. Pitfalls of masculinity contest cultures in organizations and strong single-minded work devotion in men that harm health and well-being. Underrepresentation of men in communal and HEED caring roles known to benefit self and others.	
Men as allies in gender equality progress	Considerations and consequences: Men’s attitudes towards gender equality and gender equality movements. Role of social movements related to gender-equality. Recognition of sexism.	

The knowledge reviewed here identifies effective tools to leverage change for men’s involvement, and in avoiding tools that may backfire or have negative side-effects. First, it is important that gender equality efforts are cognizant and communicate the fact that gender change is not only for women and gender-minority groups, but that gender stereotypes are a many-edged sword, negatively impacting women’s, and others’ well-being, including men and boys (Eagly et al., 2000; Croft et al., 2015; Meeussen et al., 2020; Morgenroth and Ryan, 2021). Communicating the benefits for all, and considering effects also specifically in areas where men face significant impact (e.g., health, well-being, work organizations, access to children and care for others) is more likely to generate broader support, and to reduce effects of restrictive gender roles in key areas where men face consequences. Such efforts can involve attention to gender equality in parenting, schools, the workplace, and in the media and society at large (see Croft et al., 2015; Van Laar et al., 2019; Meeussen et al., 2020 for reviews and specific recommendations). More broadly, such efforts are also likely to reduce polarization and zero-sum conflicts at the base of many political battles in societies on gender, socioeconomic status and immigration - where privileged high-status groups may focus on their own victimhood (see also Norton and Sommers, 2011; Knowles et al., 2014; Esteve et al., 2016; West, 2016; Williams, 2017; Does et al., 2018; West et al., 2021). Working as researchers, educators and practitioners we should communicate the value of gender equality efforts for all – to increase empathy and prevent zero-sum perceptions. In doing so, it is important to avoid becoming gender or color-blind with all its known downsides (i.e., focused on minimizing or ignoring differences; Richeson and Nussbaum, 2004; Dovidio et al., 2017; Gündemir et al., 2019; Leslie et al., 2020). Indeed “All-Inclusive” efforts are most likely to be successful (Stevens et al., 2008; Shih et al., 2013; Hall et al., 2018; Subašić et al., 2018). Also, we need to make clear not only how processes of disadvantage work, but also processes related to privilege and (the threat of) loss of privilege – not to assert blame, but to make explicit what often remains hidden (Schmitt and Branscombe, 2002; Pratto and Stewart, 2012; Case et al., 2014; Knowles et al., 2014; Scheepers and Ellemers, 2018; Phillips and Lowery, 2020; Hodson et al., 2022; Mikołajczak and Becker, 2022).

Many of the insights discussed are relevant not only for gender equality progress but also for other group-based inequalities, such as those based on ethnicity, social class, physical ability or sexual orientation. Indeed, allyship with other movements for equality and inclusion (such as ethnicity, SES and LGBTQ+) is key to transform norms and cultural practices. For instance, zero-sum beliefs, perceived symbolic and realistic threat, and blindness of the privileged are mechanisms that apply more generally to social systems where the historically advantaged group does not recognize the bias and discrimination against disadvantaged groups, and feels threatened by actions made towards social change (Stephan and Stephan, 2000; DiAngelo, 2011; Norton and Sommers, 2011; Pratto and Stewart, 2012). Also, many of the factors that may obstruct or aid men’s involvement in gender progress reviewed here can be applied to other social inequalities. For example, research has shown the effectiveness of empathy-inducing strategies to reduce ethnic bias (Finlay and Stephan, 2000). What does seem more specific to gender inequality is that the traditional gender framework not only disadvantages women and non-binary people, but also directly harms the wellbeing of men as the advantaged group. While lower social inequality benefits society in general and thus also the advantaged groups (Wilkinson and Pickett, 2009; Stiglitz, 2012; OECD, 2015), we have argued in this paper that men personally and directly have much to gain from gender equality.

The current paper discussed men largely as one group. In reality of course men have different ethnic, socioeconomic, religious and national identities, and different sexual and gender identities. These can affect the outlook, experiences and concerns men may have, and how the processes discussed affect them. Also, many men are not necessarily privileged themselves (e.g., in terms of ethnicity, social class, physical ability or sexual orientation - see, e.g., Coston and Kimmel, 2012; Levant and Wong, 2013; Clements et al., 2022; Goodwill et al., 2022). Moreover, much of the research has been conducted on men from WEIRD countries (Western, Educated, Independent, Rich and Democratic, Henrich et al., 2010). Nevertheless there is movement here too, with two large scale cross-national studies on gender and men’s roles with data from 62 and 49 countries, respectively, [Towards Gender Harmony project (TGH) and Understanding Communal Roles in Men project (UCOM), see Bosson et al., 2021, 2022; Kosakowska-Berezecka et al., 2022, 2024; Olsson et al., 2023; Saxler et al., 2024]. Efforts to address gender equality for men thus also need to examine the role of such differences in culture, ethnicity, religion, gender and sexual identities. Moreover, gender expectations affect men and boys at all ages – starting before birth and affecting individuals in different ways throughout their lifetimes. Taking a developmental perspective is thus also of importance (see Eckes and Trautner, 2000; Ryan and Branscombe, 2013; Way et al., 2014). Importantly, the goal of gender-related change is not to force individuals into specific nongendered roles, domains and qualities. Instead the goal is to broaden options so that choices are less driven by social constraints based on gender. Paradoxically then, in addressing DEI, we first need to focus on gender – and on men specifically – in order to in the end move away from this focus, and allow individuals to reach their potential free of gender-based restrictions. We hope that by outlining the key roles played by men in gender equality progress that we have provided some insights that aid in moving us towards this goal.

## Author contributions

CL: Conceptualization, Funding acquisition, Writing – original draft, Writing – review & editing. AR: Funding acquisition, Writing – original draft, Writing – review & editing. NK-B: Funding acquisition, Writing – original draft, Writing – review & editing. RB: Funding acquisition, Writing – original draft. KB: Writing – review & editing.

## Footnotes

^1^ www.equimundo.org

^2^ seejane.org
