# Correction: MANdatory - why men need (and are needed for) gender equality progress

**DOI:** 10.3389/fpsyg.2025.1701932

**Published:** 2025-10-03

**Authors:** Colette Van Laar, Aster Van Rossum, Natasza Kosakowska-Berezecka, Renata Bongiorno, Katharina Block

**Affiliations:** ^1^Department of Psychology, KU Leuven, Leuven, Belgium; ^2^Fonds Wetenschappelijk Onderzoek, Brussels, Belgium; ^3^Institute of Psychology, University of Gdansk, Gdansk, Pomeranian Voivodeship, Poland; ^4^School of Social Sciences, Bath Spa University, Bath, United Kingdom; ^5^Department of Psychology, University of Amsterdam, Amsterdam, Netherlands

**Keywords:** gender, social equality, social change, men and masculinity, gender roles, precarious manhood

An incorrect Funding statement was provided. The correct funder is National Science Centre in Poland.

An incorrect number was provided for National Science Centre in Poland. The correct number is 2021/41/B/HS6/00617.

The correct funding statement reads:

The author(s) declare that financial support was received for the research and/or publication of this article. This work was supported by an Odysseus grant from the Research Foundation Flanders (FWO) to CL (grant number: G.O.E66.14 N), a fellowship grant from the Research Foundation Flanders (FWO) to AR (grant number: 1114222 N), a grant from the National Science Centre in Poland (grant number: 2021/41/B/HS6/00617) awarded to NK-B, and a BA/Leverhulme Small Research Grant awarded to Renata Bongiorno as PI (grant number: SRG1920100698).

The original version of this article has been updated.

